# Infectious Mononucleosis Causing Bell’s Palsy: A Case Report

**DOI:** 10.7759/cureus.84176

**Published:** 2025-05-15

**Authors:** Roopin P Singh, William Parker, Gowrish Vaka, Abir Islam, Hassan Khanani

**Affiliations:** 1 Medicine, Edward Via College of Osteopathic Medicine, Monroe, USA; 2 Pediatrics, Willis Knighton, Shreveport, USA; 3 Internal Medicine, Edward Via College of Osteopathic Medicine, Monroe, USA

**Keywords:** bells palsy, epstein barr virus (ebv), facial nerve paralysis, infectious mononucleosis (im), kissing disease

## Abstract

Infectious mononucleosis, also called the kissing disease or “mono,” is typically caused by the Epstein-Barr virus. It is a clinical condition marked by fever, lymphadenopathy, and pharyngitis. Bell's palsy is a condition that causes sudden, temporary facial paralysis, or weakness on one side of the face. It can have various etiologies, such as congenital, neurological, infectious, neoplastic, or traumatic. This report describes the case of an 18-year-old female who was diagnosed with Bell's palsy, a rare neurological complication of Epstein-Barr virus-associated infectious mononucleosis. This instance highlights a unique manifestation of facial nerve palsy, which is atypical in cases of mononucleosis.

## Introduction

Infectious mononucleosis (IM), most often the result of Epstein-Barr virus (EBV), is a viral illness primarily affecting teenagers and young adults. It is transmitted through saliva, the reason why it is often referred to as the “kissing disease,” but it can also be passed via respiratory droplets, blood, or organ transplants. Symptoms can include a sore throat, fever, swollen lymph nodes, and extreme fatigue, as well as swelling of the liver and spleen. A heterophile antibody test is typically sufficient to diagnose it. Most people recover on their own with rest, fluids, and over-the-counter pain relievers, though, in rare cases, complications such as a ruptured spleen, neurological issues, or liver inflammation can occur. Neurological complications, such as cranial nerve palsies, Guillain-Barré syndrome, meningoencephalitis, and mononeuritis multiplex, occur in 1-5% of all patients with acute EBV infection [1]. Neurological manifestations in children may sometimes be the only clinical sign, which can sometimes delay the diagnosis.

Acute peripheral facial paralysis is the most common acute mono-neuropathy [2] and is often a challenge for physicians. Its incidence has been reported as 21.1 per 100,000 per year for children <15 years old [3]. The causes of pediatric facial nerve paralysis can be congenital or acquired, but the idiopathic form or Bell’s palsy is the most frequent (40-75% of cases) [4]. Bell's palsy is diagnosed upon the abrupt onset of unilateral facial weakness or complete paralysis of all the muscles on one side of the face, dry eye, pain around the ear, an altered sense of taste, hypersensitivity to sounds, or decreased tearing [5].

## Case presentation

An 18-year-old female with a past medical history of lower back pain and sinusitis presented to the pediatric clinic with complaints of fever, sore throat, and left-sided facial drooping. She reported noticing these symptoms one week prior. The neck pain had progressively worsened and radiated to the left ear. The patient also experienced difficulty forming words and was unable to raise her left eyebrow or smile due to facial drooping. She noted no improvement with over-the-counter medications and denied any recent travel.

On physical examination, several 1-cm mobile, tender anterior cervical lymph nodes were noted. Tonsillitis with white exudates and left ear tympanosclerosis with associated pain were observed. The left corner of the mouth was noted to droop, and the patient had difficulty making facial expressions and closing her left eye. The spleen was palpable and tender to touch. Neurological examination showed that all other cranial nerves were grossly intact. Muscle strength was 5/5 in both the upper and lower extremities, and deep tendon reflexes were present throughout. The lungs were clear to auscultation, and pulses were palpable in all extremities. No edema, cyanosis, or clubbing was noted. The skin was dry, and no rashes were observed.

Laboratory tests were ordered, including a complete blood count (CBC), rapid antigen tests for Strep, flu, and COVID-19, mononucleosis spot test, and EBV antibody profile. The patient tested negative for influenza, strep, and COVID-19. CBC revealed mild leukocytosis, but otherwise mostly normal results (Table 1). The mononucleosis spot test was positive for reactive heterophile antibodies, a hallmark finding of IM. Immunofluorescence assays showed positive IgM and IgG antibodies to EBV, with the presence of IgM antibodies indicating either a recent primary infection or a reactivated infection.

**Table 1:** Complete blood count laboratory findings *Value above normal range

Test	Result	Normal Range
White blood cells*	11.16×10³/µL	4.50–11.00×10³/µL
Lymphocytes	42.37%	20–40%
Monocytes	11.70%	2–8%
Neutrophils	45.23%	40–70%
Eosinophils	0.57%	1–6%
Basophils	0.12%	0–1%
Absolute lymphocyte count*	4.73×10³/µL	1.00–4.50×10³/µL
Absolute monocyte count*	1.31×10³/µL	0.00–1.00×10³/µL
Absolute neutrophil count	5.05×10³/µL	1.50–8.00×10³/µL
Absolute eosinophil count	0.06×10³/µL	0.00–0.50×10³/µL
Absolute basophil count	0.01×10³/µL	0.00–0.20×10³/µL
Red blood cells	4.69×10⁶/µL	3.90–5.20×10⁶/µL
Hemoglobin	12.70 g/dL	12.00–18.00 g/dL
Hematocrit	39.00%	36.0–51.0%
Mean corpuscular volume	83.2 fL	82.0–97.0 fL
Mean corpuscular hemoglobin	27.1 pg	27.0–36.0 pg
Mean corpuscular hemoglobin concentration	32.6 g/dL	31.0–36.0 g/dL
Red cell distribution width*	15.40%	10.1–14.9%
Red cell distribution width-SD	42.1 fL	35.0–56.0 fL
Platelets	332.3×10³/µL	150–400×10³/µL
Mean platelet volume	7.19 fL	5.50–24.00 fL

With these findings, a diagnosis of left-sided Bell’s palsy due to EBV infection was confirmed. The patient was prescribed prednisone 60 mg for one week, with a plan to taper the dose. Tylenol was recommended for pain management as needed. Artificial tears were prescribed to prevent corneal damage and dryness due to the inability of the left eye to close. An ear, nose, and throat (ENT) referral was made for the evaluation of left ear tympanosclerosis and associated pain. The patient was counseled to avoid contact sports due to the risk of splenic rupture, a common complication of IM.

At the two-week follow-up, the patient reported symptoms such as runny nose, fatigue, and ongoing ear pain, pending ENT doctor evaluation. At that time, there was no evidence of lymphadenopathy, fever, or splenomegaly. The patient's facial paralysis had improved by approximately 70%, and her speech was also markedly improved. The patient was then counseled to rest, hydrate, and closely monitor for any new symptoms.

## Discussion

**Figure 1:**
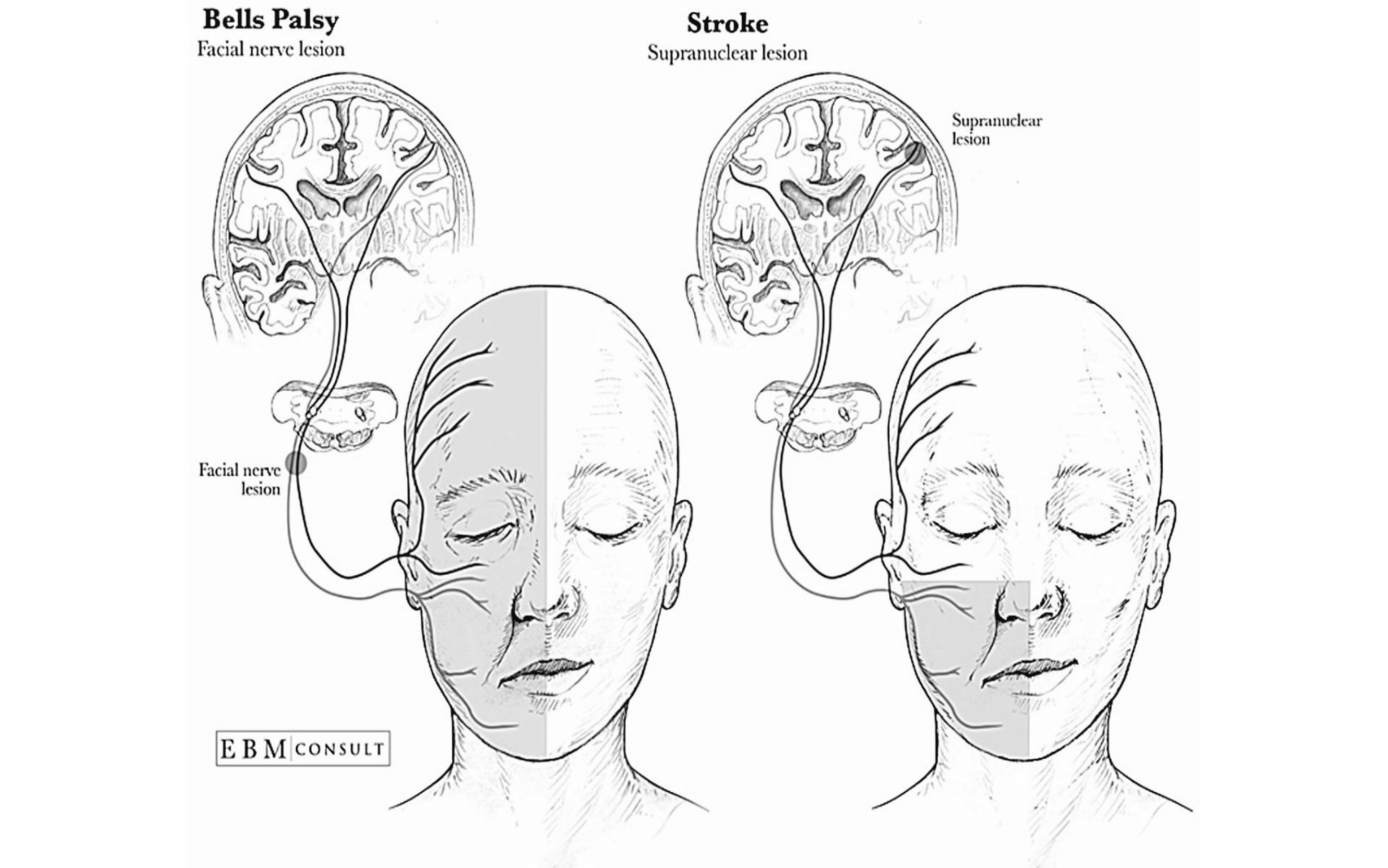
Anatomic illustration of Bell’s palsy versus stroke lesion Image provided by EBM Consult [6]

The first step in diagnosing facial weakness is to determine whether it stems from the central nervous system (CNS) or the peripheral nervous system. CNS lesions, such as those caused by multiple sclerosis, stroke, or tumors, can also lead to facial nerve palsy. Some of the motor neurons of the forehead cross over at the brainstem; therefore, forehead muscles are controlled by both sides of the brain. Consequently, if there is a lesion in the CNS, the forehead is spared, and only the lower part of the face gets affected. In contrast, Bell’s palsy causes unilateral facial paralysis, including the forehead, due to the lesion's location. A visual aid to better understand this concept of sparing is provided in Figure 1.

The frequency of peripheral facial paralysis in children is less than in adults, but the clinical diagnosis is more difficult [7]. Initial diagnosis of Bell’s palsy is usually made by ruling out other causes. Recovery time varies, but complete resolution of symptoms usually occurs after two to three weeks. There is always a small possibility of permanent functional loss, which can result from the structural damages to the axons and myelin [8]. Treatment is usually supportive, which involves a combination of steroids and physical therapy in some cases.

The origin of the condition is primarily idiopathic, often triggered by changes in microcirculation. However, it can also be congenital, caused by neoplasia or neuralgia, or result from a systemic illness. In 5% to 30% of cases, the paralysis is the result of infection, which may be viral, bacterial, or fungal (Table 2). In fact, infectious etiology is the most common origin of this condition in young children [9]. This condition can be caused by several agents, with herpes simplex virus 1 and varicella-zoster virus being the most common. Less frequently, it can be caused by other organisms such as *Borrelia burgdorferi*, Coxsackie, polio, influenza, human immunodeficiency viruses, and EBV. Early diagnosis and ruling out stroke are very crucial steps in reducing any unwanted permanent damages. Assessing for skin and recent travel to endemic areas is also crucial to rule out Lyme disease, which could also manifest as facial nerve palsy with a bull’s eye rash pattern.

**Table 2:** Causes of facial nerve paralysis Table modified from Ciorba et al. [4]

Category	Condition
Genetic	Hereditary myopathies (e.g., myotonic dystrophy, myasthenia), 3q21-22 and 10q21.3-22.1 mutations
Syndromic malformations	Congenital pseudobulbar palsy (syringobulbia), Möbius syndrome, Arnold-Chiari syndrome, Goldenhar syndrome
Delivery traumas	Prematurity, forceps, primiparity, cesarean section, birthweight > 3,500 g
Infectious	Lyme disease, Epstein-Barr virus, Ramsay Hunt syndrome, *Haemophilus influenzae*, human immunodeficiency viruses (HIV), tuberculosis, adenovirus, cytomegalovirus, *Mycoplasma pneumoniae*, mumps, rubella, chronic otitis media/cholesteatoma, acute otitis media
Inflammatory	Vasculitis, Henoch-Schönlein purpura, Kawasaki syndrome
Neoplastic	Leukemia, hemangiomas, parotid gland tumors, rhabdomyosarcoma, schwannomas of the VII cranial nerve, temporal bone histiocytosis
Trauma	Temporal bone fractures (transverse, oblique, and longitudinal)
Iatrogenic	After surgery of the mastoid, parotid gland, or middle ear
Idiopathic	Bell’s palsy

The present case exhibited all the clinical features of IM that was caused by EBV, a double-stranded DNA virus also known as human herpesvirus 4. More than half of the patients diagnosed with IM present with a triad of fever, lymphadenopathy, and pharyngitis [10]. Laboratory studies often reveal thrombocytopenia, leukocytosis, elevated aminotransferases, and the presence of heterophile antibodies [10]. Neurologic complications during the post-infectious state of IM have been rarely reported. The initial case reports date back to the early thirties [11]. Like other herpetic viruses, EBV is a lifelong infection that exhibits different patterns at different stages of life. The first antibodies generated during the course of primary EBV infection are against the viral capsid antigen (VCA) complex. The IgM antibodies are transient, and the IgG antibodies persist for life [12]. In our case, immunofluorescence assays of antibody titers to EBV antigens showed the presence of VCA IgM and IgG antibodies, indicating an acute primary EBV infection.

There are some potential mechanisms by which Bell’s palsy can develop from IM. The EBV may directly invade the facial nerve, leading to inflammation and triggering immune responses that can cause nerve damage. Additionally, lymphoid hyperplasia and swelling of nearby structures, such as the parotid gland, can compress the facial nerve [6]. EBV reactivation in latent B cells and vascular compromise due to vasculitis can impair blood supply to the facial nerve. All these mechanisms can collectively contribute to facial nerve dysfunction.

Although facial nerve palsy has been reported as a complication of IM, this association remains underrecognized by clinicians worldwide. EBV has been proposed in the literature as a potential cause of peripheral facial paralysis, though definitive cases in both pediatric and adult patients remain rare. This case study, therefore, provides valuable insight into EBV as an underlying cause of facial paralysis, contributing to the existing body of knowledge on the topic.

## Conclusions

This case presents a unique neurological complication of IM caused by EBV. While the exact cause remains unclear, inflammation and vascular compromise of the facial nerve are suspected as potential mechanisms. Since this complication is uncommon, it is often underdiagnosed or mistaken for other causes of facial paralysis, potentially resulting in delayed treatment and unnecessary interventions. Prompt recognition of this rare association is essential to ensure appropriate treatment and prevent any lifelong complications.
